# Association of parental methylenetetrahydrofolate reductase (MTHFR) C677T gene polymorphism in couples with unexplained recurrent pregnancy loss

**DOI:** 10.1186/s13104-018-3321-x

**Published:** 2018-04-05

**Authors:** Anil Kumar Sah, Nisha Shrestha, Pratikshya Joshi, Renu Lakha, Sweta Shrestha, Laxmi Sharma, Avinash Chandra, Neetu Singh, Yuvraj KC, Bhola Rijal

**Affiliations:** 1 Annapurna Research Center, Maitighar, Kathmandu, Nepal; 2 SANN International College, Gairidhara, Kathmandu, Nepal; 3 Annapurna Neurological Institute and Allied Sciences, Maitighar, Kathmandu, Nepal; 4 Om Hospital and Research Center, Chabahil, Kathmandu, Nepal

**Keywords:** Methylenetetrahydrofolate reductase (MTHFR), PCR–RFLP, Recurrent pregnancy loss, Nepal

## Abstract

**Objective:**

The aim of this study was to identify the association of parental MTHFR C677T gene polymorphism in couples with and without RPL history.

**Results:**

During the study, 21.4% (15/70) of Ala222Val polymorphism was observed among RPL couples while no polymorphism was seen among normal, healthy couples. Our study did not find any association between MTHFR C677T polymorphism and gender (p > 0.05), gestational period (p > 0.05), geographical region (p > 0.05) and menstrual history (p > 0.05). However, significant association was seen between MTHFR C677T polymorphism and number of losses (p < 0.05), concluding that the risk of the polymorphism increased with the increase in number of losses. Significant variation in the MTHFR C677T genotype with number of losses among RPL couples were seen but not with other study variables.

**Electronic supplementary material:**

The online version of this article (10.1186/s13104-018-3321-x) contains supplementary material, which is available to authorized users.

## Introduction

Recurrent pregnancy loss (RPL) is defined as two or more consecutive miscarriages before 20 weeks’ of gestation. It is a multifactorial disorder like genetic disorders, endocrine dysfunctions, uterine pathologies, autoimmune diseases, acquired and inherited thrombophilia as well as environmental factors are major concern in gynecology [1–4]. Various studies showed that the inherited thrombophilic polymorphisms are significant risk factors for pre-eclampsia, placental abruption, stillbirth and fetal growth restriction [5, 6]. Karyotyping showed that parental chromosomal abnormalities occur in one of the partners in 5–7% of couples who suffer from RPL out of which half of the cases remain unexplained [7]. Currently, there are only limited data on a possible male cause.

Recurrent pregnancy loss is also considered to be associated with inherited thrombophilia that take in diverse conditions including the thermolabile variation of the 5,10-methylenetetrahydrofolate reductase (MTHFR) and the mutation is also associated with hyperhomocysteinemia. The enzyme MTHFR plays a critical role in the folate metabolism pathway, and regulates the intracellular folate pool for synthesis and methylation of DNA [8, 9].

MTHFR C667T mutation have been described in Brazilian [9], Japanese [10], Indian [11], and Sri Lankan [12]. Since Nepal shares the ancestral origin with India and people have been sharing similar lifestyles for a long period of life it was relevant to check the occurrence of same mutation in RPL population of Nepal as well. According to Nepal demographic and health survey (2011) there is 7% of population having unknown causes of miscarriages and there are so many causes behind it. Therefore the study was designed with the aim to identify the association of parental MTHFR C677T gene polymorphism in couples with RPL in Nepalese population.

## Main text

### Methods

Hospital based cross-sectional study was conducted at Annapurna Research Center, Kathmandu and other tertiary care hospital of Kathmandu, Nepal from September 2016 to March 2017. The study populations were both inpatients, and outpatients with RPL cases attending Obstetrics and Gynecology (OB/GYN) department of different hospitals in Kathmandu valley. A total of 70 couples were enrolled in the study; out of which 35 couples were having RPL and 35 were healthy.

#### Inclusion and exclusion factors for sample selection

Recurrent pregnancy loss couples with two or more consecutive miscarriages with or without normal child, unexplained cause of losses and for normal healthy couples with at least one or more live births without any history of abortion were included in the study. RPL couples with known cause of losses like accidents, hormonal imbalances, blood grouping factors, chromosomal abnormalities (if karyotyping is done) uterine anomalies, genital infections, and endocrinological disorders were excluded from the study. Signed informed consent was obtained from all patients prior to the study.

#### DNA extraction and thrombophilic mutation

Blood samples from both normal and RPL subjects were collected. DNA was extracted using Wizard^®^ Genomic DNA Purification kit (Promega Corporation, US). Spectrophotometric analysis was carried out to check the quality and quantity of DNA samples. Genotype screening was performed for identification of MTHFR gene among 70 couples.

The MTHFR C677T gene of 198 bp was amplified by polymerase chain reaction (PCR) using oligonucleotide primer sequences forward 5′-TGAAGGAGAAGGTGTCTGCGGGA-3′ and reverse 5′-AGGACGGTGCGGTGAGAGTG-3′ (Macrogen, Korea). The PCR was carried out on 25 μl reaction mixture of 12.5 μl master mix (Solis BioDyne, Estonia), 0.5 μl primers (10 pmol/μl), 3 µl of DNA was added while equal amount DNA of positive control and PCR grade water were added as negative controls then final volume of PCR-mix was adjusted by adding PCR grade water in above solution. The PCR tubes were placed on the tube holder of the thermo-cycler (Techne, UK). The cycle parameters for PCR initial denaturation at 94 °C for 10 min followed by 35 amplification cycles of denaturation at 94 °C for 1 min; annealing at 60 °C for 1 min and extension at 72 °C for 1 min: followed by the final extension reaction at 72 °C for another 7 min. For the confirmation of PCR amplification 2% agarose gel was prepared in TBE (1×) buffer and ethidium bromide (EtBr-10 mg/ml) was added to it. The agarose gel was run at 80 V for 40 min. Then, agarose gel was visualized under gel doc system (UV Cambridge, USA).

#### Restriction fragment length polymorphism (RFLP)

**Fig. 1:**
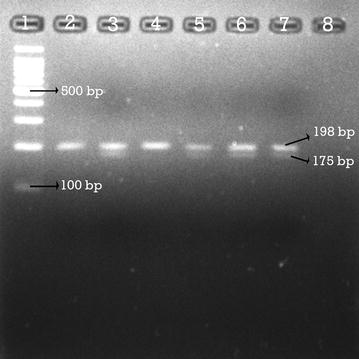
RFLP analysis after digestion with HinfI showing homozygous C/C, heterozygous C/T genotype variants. Lane 2, 4: Homozygous CC wild type which is normal. Lane 3, 5, 6, 7: Heterozygous C/T mutation. Lane 1: The 100 bp DNA ladder

**Fig. 2:**
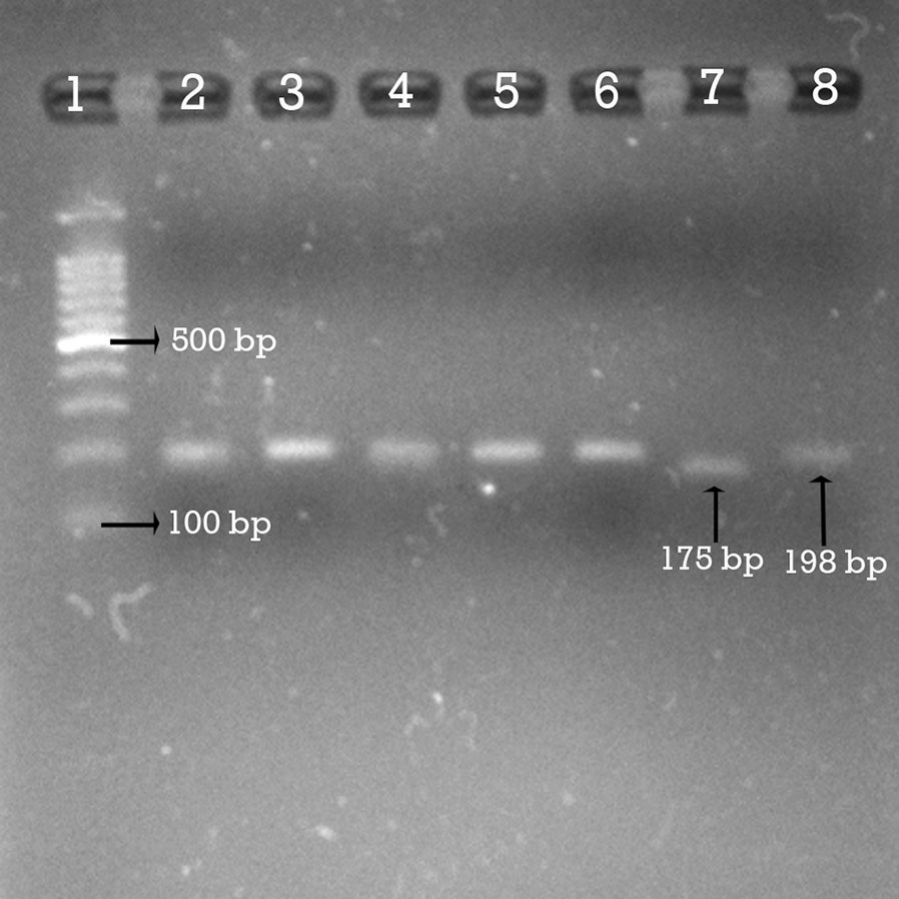
RFLP analysis after digestion with HinfI showing homozygous C/C, homozygous T/T genotype variants. Lane 2, 3, 4, 5, 6, 8: Homozygous CC wild type which is normal. Lane 7: Homozygous T/T mutation. Lane 1: The 100 bp DNA ladder

Genotyping C677T polymorphism was done following PCR/HinfI restriction digestion (Promega Corporation, US). A change in HinfI restriction-site at position 677, where C is replaced by T indicates the presence of the mutation. The amplified PCR products were digested with 5U HinfI restriction enzyme (Promega, USA) in 20 μl reaction volume contained; PCR Product (1 µg/µl) 1.5 µl, Buffer B (10×) 2 μl, Acetylated (BSA) (10 µg/µl) 0.2 μl, Restriction enzyme (HinfI) (10 unit/µl) 0.5 μl, Nuclease free water 15.8 μl then incubated at 37 °C for 4 h. The digested DNA fragments were separated by agarose gel-electrophoresis in 3% agarose gel, run at 60 V for 1 h and 30 min and the bands were then examined under a UV light of gel doc system (UV Cambridge, USA) as shown in (Figs. 1, 2).

#### Statistical analysis

Genotype distributions of each polymorphism, percentage and frequency of hetero and homozygocity were compared between the couples with RPL. Pearson’s Chi square test was used to assess intergroup significance at 95% confidence interval.

### Results

Samples were collected from 35 normal couples and 35 couples with RPL from different hospitals in Kathmandu valley. The MTHFR polymorphism was seen on 21.42% (15/35) couples with RPL and was not seen in any of the normal couples (Table 1). Accordingly, the frequency of C677T genotype MTHFR gene in RPL couples showed homozygous wild type CC in 77.2% (27/35) in male and 80% (28/35) in female, heterozygous CT type in 20% (7/35) in male and 14.3% (5/35) in female and homozygous mutation TT type in 2.8% (1/35) in male and 5.7% (2/35) in female (Additional file 1: Table S1). There was not any significant difference of the TT genotype between male and female groups (p = 0.71). The distribution of MTHFR C677T polymorphism was more prevalent in the RPL cases with 3 losses than the cases with 2 and 4 losses and there was significant relationship between number of losses and polymorphism (p = 0.033). Increased prevalence of MTHFR C677T polymorphism was seen in 1st trimester and 2nd trimester, but not in 3rd trimester (p = 0.138). MTHFR C677T polymorphism was seen more in age group of 21–30 years (12.85%) than in age group of 31–40 years (8.5%) (p = 0.28).

**Table 1:** The genotype distribution of each MTHFR polymorphism in RPL and normal couples

Type	No. of samples	Non-polymorphic	polymorphic
Normal	70	70 (100%)	0 (0%)
Patients with RPL	70	55 (78.6%)	15 (21.42%)
Total	140	125 (89.3)	15 (10.7%)

### Discussion

There are many controversies regarding MTHFR C677T gene polymorphism and miscarriage, some studies supported the direct evidence between C677T gene mutation and RPL [7, 12, 13].

Out of 70 samples of RPL couples, polymorphism was seen in 15 (21.4%) of them. Hence, our study found a significant role of MTHFR C677T polymorphism in RPL, a finding that is in concordance with a previous study conducted by Mtiraoui et al. [13] out of 200 patients, 108 had a mutation in the MTHFR gene in RPL patients and 44 in control group. However in this study no homozygous C677T mutation was seen in the 35 healthy couples that might be due to control group from single community of Newar.

According to Van der Molen el al [14], homozygous C677T mutation (TT genotype) was observed in 12% of women in the study group (n = 19/165) and 5% (n = 7/139) in the control group. Similarly, a study conducted in North Indian women reported that RPL was correlated with MTHFR variant genotype (p < 0.01) [15, 16]. It also stated that the presence of MTHFR polymorphism significantly increased the risk of RPL by more than three fold. However, a study conducted in Palestinian women, there was no statistical difference among the allele distribution and the genotype frequencies between case and control group (p > 0.05) [17, 18]. Likewise, meta-analysis among the Dutch women homozygosity for a common 677 C → T mutation in MTHFR gene, leads to a two to threefold higher risk of RPL [19]. Hence, in general homozygosity for the mutated MTHFR gene is a risk factor for RPL.

Homozygosity for MTHFR C677T polymorphism is reported to be a maternal risk factor for RPL. Little is known about the role of paternal risk factors in RPL. The current study was undertaken to examine the association between both maternal and paternal MTHFR C677T polymorphism and RPL. Polymorphism was observed 22.9% (8/35) in male, while 20% (7/35) in female.

In two RPL couples, we observed heterozygous CT mutation in both male and female. The obstetrics history reveals that they had three spontaneous abortions. Since the MTHFR enzyme deficiency is an autosomal recessive type, when both parents are carrying mutant allele the chances of inheritance to the offspring is much higher which may increase the risk of abortion by increasing the homocysteine levels in mother as well as in the fetus and thereby increasing thrombotic activity at the feto-placental interphase [12]. Our study did not find any association between gender and MTHFR C677T polymorphism (p > 0.05), which was similar to the finding of the study of Van der Molen and colleague [15]. They had also reported that there was no any prevalent difference of the TT genotype between Dutch men and women (p > 0.05).

The distribution of MTHFR C677T polymorphism was seen in cases with 2, 3 and 4 losses respectively. This study showed association between number of losses and MTHFR C677T gene polymorphism was significant (p < 0.05), as in meta-analysis [19] in which women with three or more miscarriages had a much higher risk of RPL than hyperhomocysteinemic women with only two miscarriages. The possible reason could be that women with three or more pregnancy losses had lower median serum folate levels [20]. However, Abu-Asab and colleague did not find a significant association between MTHFR and RPL in the first and second trimesters [18]. Likely, in our study too, the association between MTHFR and RPL was not significant in the second and third trimesters (p > 0.05); though, the association was seen in first trimester (n = 9/70). This might be due to the use of folic acid among the pregnant women, especially during the first trimester. Folate level plays a significant role in regulating homocysteine level in individuals homozygous for MTHFR. Therefore, it was reasonable to assume that folic acid consumption may effectively reduce the adverse effects of MTHFR and decrease the risk of RPL [21].

### Conclusions

The findings of this study show significant association between MTHFR C677T genotype with number of losses among RPL couples and not with other variables such as age, gender and gestational period. The MTHFR C677T polymorphism was seen in RPL group and variations were found in the genotype distribution among men with RPL impact. In accordance to findings of this study, heterozygous variant in both male and female suggests that paternal screening is equally essential along with maternal screening.

## Limitation

Paternal screening along with maternal screening is needed to be screened for polymorphism. Concentration of folate, homocysteine and vitamin B12 in pregnant women should be evaluated. Study with a larger population of RPL and normal couples should be conducted.

## Additional file

**Additional file 1: Table S1.** Showing MTHFR C677T Genotypes.

## Abbreviations

RPL: recurrent pregnancy loss
MTHFR: methylenetetrahydrofolate reductase
PCR: polymerase chain reaction

## References

[1] Alijotas-Reig J, Garrido-Gimenez C (2013). Current concepts and new trends in the diagnosis and management of recurrent miscarriage. Obstet Gynecol Surv.

[2] Ural ÜM, Tekin YB, Balik G, Şahin FK, Çolak S (2014). Could platelet distribution width be a predictive marker for unexplained recurrent miscarriage?. Arch Gynecol Obstet.

[3] Cramer DW, Wise LA: The epidemiology of recurrent pregnancy loss. In: Seminars in reproductive medicine: 2000: Copyright© 2000 by Thieme Medical Publishers, Inc., 333 Seventh Avenue, New York, NY 10001, USA. Tel.: + 1 (212) 584-4662; 2000: 331-340. 10.1055/s-2000-13722 11355791

[4] Parazzini F, Bocciolone L, Fedele L, Negri E, La Vecchia C, Acaia B (1991). Risk factors for spontaneous abortion. Int J Epidemiol.

[5] Kupferminc M, Fait G, Many A, Gordon D, Eldor A, Lessing J (2000). Severe preeclampsia and high frequency of genetic thrombophilic mutations. Obstet Gynecol.

[6] Brenner B, Sarig G, Weiner Z, Younis J, Blumenfeld Z, Lanir N (1999). Thrombophilic polymorphisms are common in women with fetal loss without apparent cause. Thromb Haemost.

[7] Ueland PM, Hustad S, Schneede J, Refsum H, Vollset SE (2001). Biological and clinical implications of the MTHFR C677T polymorphism. Trends Pharmacol Sci.

[8] Boers G (1997). Hyperhomocysteinemia as a risk factor for arterial and venous disease. A review of evidence and relevance. Thromb Haemost.

[9] Amarakoon AGU, Fernandopulle N (2016). Detection of C677T & A1298C mutations within the MTHFR gene by PCR and RFLP assays and assessment of risk factor of Hyperhomocysteinemia. World Sci News.

[10] Boas WV, Gonçalves RO, Costa OLN, Goncalves MS (2015). Metabolism and gene polymorphisms of the folate pathway in Brazilian women with history of recurrent abortion. Revista Brasileira de Ginecologia e Obstetrícia.

[11] Makino A, Nakanishi T, Sugiura-Ogasawara M, Ozaki Y, Suzumori N, Suzumori K (2004). No association of C677T methylenetetrahydrofolate reductase and an endothelial nitric oxide synthase polymorphism with recurrent pregnancy loss. Am J Reprod Immunol.

[12] Vanilla S, Dayanand C, Kotur PF, Kutty MA, Vegi PK (2015). Evidence of paternal N5, N10-methylenetetrahydrofolate reductase (MTHFR) C677T gene polymorphism in couples with recurrent spontaneous abortions (RSAs) in Kolar District—a South West of India. J Clin Diagn Res JCDR.

[13] Mtiraoui N, Zammiti W, Ghazouani L, Braham NJ, Saidi S, Finan R, Almawi W, Mahjoub T (2006). Methylenetetrahydrofolate reductase C677T and A1298C polymorphism and changes in homocysteine concentrations in women with idiopathic recurrent pregnancy losses. Reproduction.

[14] Van der Molen EF, Arends GE, Nelen WL, van der Put NJ, Heil SG, Eskes TK, Blom HJ (2000). A common mutation in the 5,10-methylenetetrahydrofolate reductase gene as a new risk factor for placental vasculopathy. Am J Obstet Gynecol.

[15] Tiwari D, Bose PD, Das S, Das CR, Datta R, Bose S (2015). MTHFR (C677T) polymorphism and PR (PROGINS) mutation as genetic factors for preterm delivery, fetal death and low birth weight: a Northeast Indian population based study. Meta Gene.

[16] Parveen F, Tuteja M, Agrawal S (2013). Polymorphisms in MTHFR, MTHFD, and PAI-1 and recurrent miscarriage among North Indian women. Arch Gynecol Obstet.

[17] Cardona H, Castañeda SA, Cardona Maya W, Alvarez L, Gómez J, Gómez J, Torres J, Tobón L, Bedoya G, Cadavid ÁP (2012). Lack of association between recurrent pregnancy loss and inherited thrombophilia in a group of Colombian patients. Thrombosis.

[18] Nelen WL, Bulten J, Steegers EA, Blom HJ, Hanselaar AG, Eskes TK (2000). Maternal homocysteine and chorionic vascularization in recurrent early pregnancy loss. Hum Reprod.

[19] Abu-Asab N, Ayesh S, Ateeq R, Nassar S, El-Sharif W (2011). Association of inherited thrombophilia with recurrent pregnancy loss in palestinian women. Obstet Gynecol Int.

[20] Nelen WL, Blom HJ, Steegers EA, Den Heijer M, Thomas CM, Eskes TK (2000). Homocysteine and folate levels as risk factors for recurrent early pregnancy loss. Obstet Gynecol.

[21] Yousefian E, Kardi MT, Allahveisi A (2014). Methylenetetrahydrofolate reductase C677T and A1298C polymorphism in Iranian women with idiopathic recurrent pregnancy losses. Iranian Red Cresc Med J.

