# Supplementary material for: Association of parental methylenetetrahydrofolate reductase (MTHFR) C677T gene polymorphism in couples with unexplained recurrent pregnancy loss
Source: BMC Res Notes. 2018 Apr 5;11:233. doi: 10.1186/s13104-018-3321-x (PMC5887178; doi:10.1186/s13104-018-3321-x)
Supplement: Supplementary file 1 — Additional file 1: Table S1. Showing MTHFR C677T Genotypes. [file 13104_2018_3321_MOESM1_ESM.docx]

**Table S1: Showing MTHFR C677T Genotypes**

| **Variable** |  | **Polymorphism** | | | **Total** | **P-value** |
| --- | --- | --- | --- | --- | --- | --- |
|  |  | Homozygous Rare TT | Heterozygous CT | Homozygous Wild CC |  |  |
| **Gender** | Male | 1 (2.8%) | 7 (20%) | 27 (77.2%) | 35 (100%) | 0.710 |
|  | Female | 2 (5.7%) | 5(14.3%) | 28(80%) | 35 (100%) |  |
| **No. of losses** | Two | 2 (2.8%) | 2 (2.8%) | 30 (42.8%) | 34 (48.6%) | 0.033 |
|  | Three | 0 | 9 (12.9%) | 17 (24.3%) | 26 (37.1%) |  |
|  | Four | 1(1.4%) | 1(1.4%) | 8 (11.4%) | 10 (14.3%) |  |
| **Gestation Period** | 1^st^ | 3 (4.3%) | 6 (8.6%) | 42 (60.0%) | 51 (72.9%) | 0.138 |
|  | 2^nd^ | 3 (4.3%) | 6 (8.6%) | 11 (15.7%) | 17 (24.3%) |  |
|  | 3^rd^ | 0 | 0 | 2 (2.9%) | 2 (2.8%) |  |
| **Age** | 21-30 | 3 (4.3%) | 6 (8.6%) | 31(44.3%) | 40 (57.1%) | 0.285 |
|  | 31-40 | 0 | 6 (8.6%) | 24 (34.3%) | 30 (42.9%) |  |
